# Update on Mechanisms of Renal Tubule Injury Caused by Advanced Glycation End Products 

**DOI:** 10.1155/2016/5475120

**Published:** 2016-02-29

**Authors:** Hong Sun, Yang Yuan, Zilin Sun

**Affiliations:** Department of Endocrinology, Zhongda Hospital, Institute of Diabetes, Medical School, Southeast University, Nanjing 210009, China

## Abstract

Diabetic nephropathy (DN) caused by advanced glycation end products (AGEs) may be associated with lipid accumulation in the kidneys. This study was designed to investigate whether N*ε*-(carboxymethyl) lysine (CML, a member of the AGEs family) increases lipid accumulation in a human renal tubular epithelial cell line (HK-2) via increasing cholesterol synthesis and uptake and reducing cholesterol efflux through endoplasmic reticulum stress (ERS). Our results showed that CML disrupts cholesterol metabolism in HK-2 cells by activating sterol regulatory element-binding protein 2 (SREBP-2) and liver X receptor (LXR), followed by an increase in 3-hydroxy-3-methylglutaryl coenzyme A reductase (HMG-CoAR) mediated cholesterol synthesis and low density lipoprotein receptor (LDLr) mediated cholesterol uptake and a reduction in ATP-binding cassette transporter A1 (ABCA1) mediated cholesterol efflux, ultimately causing lipid accumulation in HK-2 cells. All of these responses could be suppressed by an ERS inhibitor, which suggests that CML causes lipid accumulation in renal tubule cells through ERS and that the inhibition of ERS is a potential novel approach to treating CML-induced renal tubular foam cell formation.

## 1. Introduction

Diabetic nephropathy (DN) is the most serious complication of diabetes mellitus and the leading cause of end-stage renal disease (ESRD) [1]. DN not only involves vascular and glomerular changes but also has a particularly important relationship with tubular metabolism, structure, and function. Many factors are related to the pathogenesis of diabetic renal tubule injury, such as advanced glycation end products (AGEs) [2]. AGEs are nonenzymatic protein glycosylation products caused by glucose-induced metabolic disorder and play an important role in diabetes and its complications [3]. N*ε*-(carboxymethyl) lysine (CML) is one of the major AGEs* in vivo* [4], and the level of CML is elevated in the serum and organs of diabetic patients [5–8]. During DN progression, CML binds to a specific receptor for AGEs (RAGE) expressed on the surface of tubule epithelial cells and activates intracellular signaling pathways, thereby exerting multiple biological effects [9].

The endoplasmic reticulum (ER) is a membranous network that is involved in the synthesis and processing of secretory and membrane-bound proteins. A number of pathological stress conditions can disrupt ER homeostasis and trigger ER stress (ERS), leading to abnormal protein structure and function in the ER. Sterol regulatory element-binding protein 2 (SREBP-2), an isoform of SREBPs, is synthesized as an inactive form that is bound to the ER in a complex with SREBP cleavage-activating protein (SCAP) [10]. SCAP shuttles SREBP-2 from the ER to the Golgi, where SREBP-2 is cleaved by two proteases (site 1 and site 2 proteases). Then, the cleaved N-terminal fragment (nSREBP-2) enters the nucleus, binds to the sterol regulatory elements in the promoters of low density lipoprotein receptor (LDLr) and 3-hydroxy-3-methylglutaryl coenzyme A reductase (HMG-CoAR), and upregulates the transcription of these genes, resulting in increases in cholesterol uptake and synthesis [11–13]. There is a delicate system that regulates cholesterol homeostasis and maintains a balance between the uptake, synthesis, and disposal of cholesterol in all cells. Liver X receptor (LXR) plays a key role in maintaining cholesterol homeostasis and the transport of cholesterol. In particular, LXR activates the expression of ATP-binding cassette transporter A1 (ABCA1) [14–16], which accelerates cholesterol efflux, resulting in the formation of high density lipoprotein (HDL), and promotes reverse cholesterol transport (RCT) from peripheral tissues to the liver [17].

Our previous studies have discovered lipid accumulation in the renal tubules of type 2 diabetic rats [18], but the mechanisms responsible for this phenomenon are still unknown. The present study explores whether the lipid deposition in renal tubule cells is caused by AGEs-triggered ERS.

## 2. Materials and Methods

### 2.1. Cell Culture

The human renal tubule epithelial cell line HK-2 (a gift from Dr. BC Liu) was cultured with Dulbecco's Modified Eagle's Medium/Ham's Nutrient Mixture F-12 (DMEM-F12) containing 10% fetal bovine serum. All experiments were performed in serum-free DMEM-F12 medium containing 0.2% BSA, 100 U/mL penicillin, and 100 *μ*g/mL streptomycin. The reagents used for cell culture were obtained from HyClone (Logan, Utah, USA). CML, which was produced via organic synthesis (no material of animal or human origin used), was obtained from Santa Cruz (Delaware Avenue, USA). 4-Phenyl butyric acid (4-PBA), purchased from Sigma (California, USA), was used to inhibit ERS [19, 20]. An anti-RAGE antibody, which was used to block the CML-RAGE pathway [21], was obtained from R&D Systems (Minneapolis, MN, USA).

### 2.2. Cell Viability Assay

Cell viability was determined using CCK-8 dye (Yiyuan Biotechnologies, China) according to the manufacturer's instructions. In brief, 3 × 10^3^ cells/well were seeded in a 96-well plate and cultured at 37°C for 24 h. After a starvation period, HK-2 cells were incubated for 24 h in experimental medium (Ctr), in the presence of 50 *μ*g/mL CML, 50 *μ*g/mL CML plus 10 *μ*g/mL anti-RAGE antibody (CML + anti-RAGE), and 50 *μ*g/mL CML plus 5 mM 4-PBA (CML + 4-PBA) or in the presence of 4-PBA alone. After 10 *μ*L of CCK-8 dye was added to each well, the cells were incubated at 37°C for 2 h, and the absorbance was then determined at 450 nm using a microplate reader.

### 2.3. Cell Apoptosis Assay

Cell apoptosis was determined using the Annexin V-FITC Kit (Biouniquer Technology, China) according to the manufacturer's instructions. In brief, 10^6^ cells/well were seeded in a 6-well plate and cultured at 37°C for 24 h. After a starvation period, the cells were treated under different experimental conditions for 24 h. Then, after the addition of 100 *μ*L of Binding buffer, 10 *μ*L of Annexin V-FITC, and 5 *μ*L of a Propidium Iodide (PI) solution, the cells were incubated at 37°C for 10 min and subsequently analyzed by flow cytometry. The double staining of cells with Annexin V-FITC and PI permits the identification of different cell populations based on their staining patterns as follows: live (FITC−PI−), early apoptotic (FITC+PI+), late apoptotic (FITC+PI−), and necrotic cells (FITC−PI+).

### 2.4. Observation of Lipid Accumulation

Lipid accumulation in HK-2 cells was evaluated with Oil Red O staining. Briefly, after treatment, samples were fixed with 4% paraformaldehyde and then stained with Oil Red O for 30 min. Finally, the samples were counterstained with hematoxylin for 5 min. The results were examined by light microscopy.

### 2.5. Quantitative Measurement of Intracellular Cholesterol

HK-2 cells in six-well plates were cultured for 24 h under different experimental conditions. The cells were then washed twice in PBS, and the total cholesterol (TC) and free cholesterol (FC) contents were measured with enzymatic assays (Applygen Technologies Inc., Beijing, China). The concentration of cholesterol ester (CE) was calculated using the equation TC − FC.

### 2.6. RNA Extraction and Real-Time PCR

Total RNA was isolated from cultured HK-2 cells using TRIzol (Ambion, Huntingdon, UK). Then, RNA (1 *μ*g) was used as a template for RT with a High Capacity cDNA RT Kit from ABI (Applied Biosystems, Warrington, UK). Real-time RT-PCR was performed in an ABI 7000 Sequence Detection System using SYBR Green Dye according to the manufacturer's protocol (Applied Biosystems). All the PCR primers were synthesized by Jierui Biotechnology (Shanghai, China). The sequences and the amplified lengths are shown in Table 1.

### 2.7. Protein Extraction and Western Blot Analysis

Identical amounts of protein from whole-cell and nuclear extracts were denatured and then subjected to electrophoresis on 10% SDS polyacrylamide gels. Proteins were transferred to a polyvinylidene fluoride membrane (Millipore Corporation, Bedford, MA, USA), which was then blocked for 1 h at room temperature with 5% bovine serum albumin in Tris-buffered saline containing 0.05% Tween 20 (TBST). Subsequently, the blots were washed and incubated overnight at 4°C in TBST containing 5% bovine serum albumin with a 1 : 1000 dilution of antibodies directed against HMG-CoAR, LDLr, SREBP-2, LXR*α*, ABCA1, 78-kDa glucose-regulated protein (GRP78), C/EBP homologous protein (CHOP), and *β*-actin (Abcam, Cambridge, UK). The rabbit anti-SREBP-2 antibody can detect both the precursor segment and mature segment of the SREBP-2 protein. The membranes were washed three times with TBST, incubated with a secondary antibody (1 : 5000 dilution in TBST containing 5% bovine serum albumin; Santa Cruz Biotechnology) for 120 min at room temperature, and then washed three times with TBST. After the chemiluminescence reaction (Pierce, Rockford, IL, USA), bands were detected by exposing the blots to X-ray film for the appropriate time period. For a quantitative analysis, the bands were detected and evaluated densitometrically with LabWorks software (UVP Laboratory Products, Upland, CA, USA) and normalized to the density of *β*-actin or histone-H bands.

### 2.8. Statistics

All experiments were repeated at least three times. In all experiments, the data were expressed as the means ± SD and analyzed using SPSS 18.0 for Windows. The means of every pair of data sets were determined with Student's* t*-test. *P* < 0.05 was considered to be statistically significant.

## 3. Results

### 3.1. Effect of CML on Cell Viability and Apoptosis

**Figure 1:**
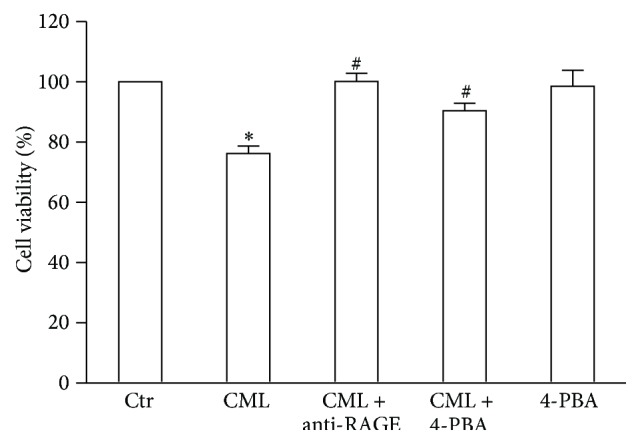
Effects of CML, anti-RAGE antibody, and an ERS inhibitor on HK-2 cell viability. Cell viability was determined after incubation with CML, CML plus anti-RAGE antibody, CML plus 4-PBA, or 4-PBA alone for 24 h. The data are expressed as the means ± SD (*n* = 3). ^*∗*^ *P* < 0.05, compared with the Ctr; ^#^ *P* < 0.05, compared with CML.

**Figure 2:**
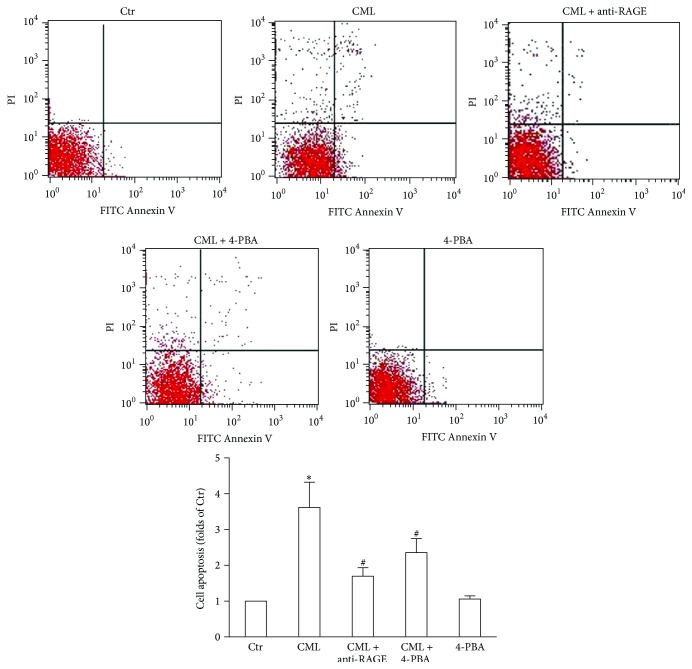
Effects of CML, anti-RAGE antibody, and an ERS inhibitor on HK-2 cell apoptosis. Cell apoptosis was determined after incubation with CML, CML plus anti-RAGE, CML plus 4-PBA, or 4-PBA alone for 24 h. HK-2 cell apoptosis includes early apoptotic (FITC+PI−) and late apoptotic (FITC+PI+) cells. The histogram represents the means ± SD of the numbers of apoptotic cells from 3 experiments, expressed as a percentage of the Ctr. ^*∗*^ *P* < 0.05, compared with the Ctr; ^#^ *P* < 0.05, compared with CML.

Cell viability was evaluated with the CCK-8 assay, and cell apoptosis was evaluated with the Annexin V-FITC Kit. Compared with the control, decreased cell viability and increased cell apoptosis were observed in HK-2 cells after CML treatment for 24 h. However, these effects could be inhibited by an anti-RAGE antibody (Figures 1 and 2).

### 3.2. Effect of CML on Intracellular Cholesterol Content

**Figure 3:**
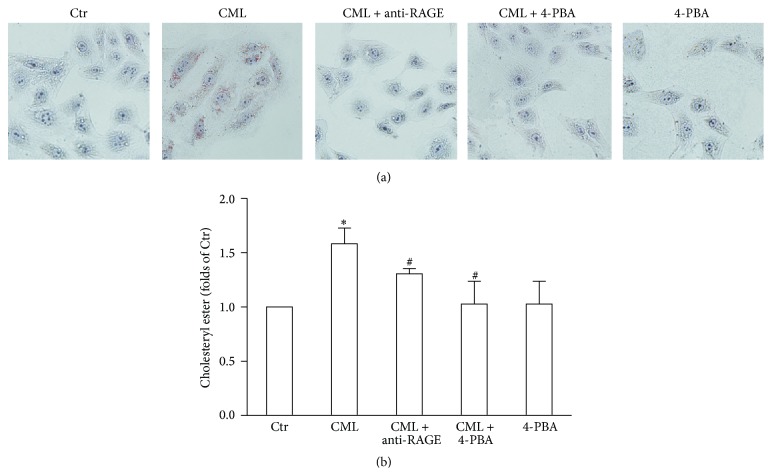
Effects of CML, anti-RAGE antibody, and an ERS inhibitor on the intracellular cholesterol content of HK-2 cells. Oil Red O staining (a) and intracellular cholesterol ester (b) were examined after incubation with CML, CML plus anti-RAGE antibody, CML plus 4-PBA, or 4-PBA alone for 24 h. The histogram represents the means ± SD of the intracellular cholesterol ester content from 3 experiments, expressed as a percentage of Ctr. ^*∗*^ *P* < 0.05, compared with the Ctr; ^#^ *P* < 0.05, compared with CML.

We assessed lipid accumulation in HK-2 cells in response to CML. The results showed that CML increased Oil Red O staining and the level of intracellular cholesterol ester in HK-2 cells. Compared with the CML-treated cells, the anti-RAGE antibody obviously attenuated lipid deposition in renal tubule epithelial cells (Figures 3(a) and 3(b)).

### 3.3. Effect of CML on Cholesterol Uptake, Synthesis, and Efflux

**Figure 4:**
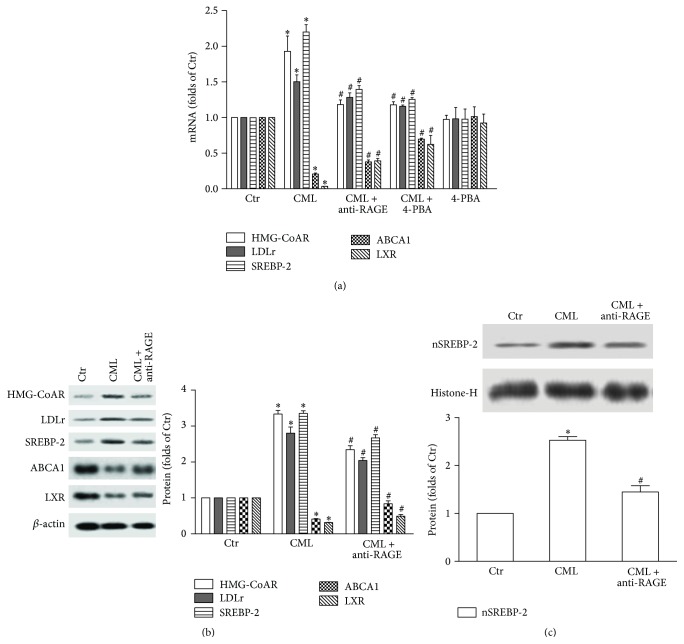
Effects of CML on the mRNA and protein expression levels of HMG-CoAR, LDLr, SREBP-2, ABCA1, and LXR in HK-2 cells. After incubation with CML, CML plus anti-RAGE antibody, CML plus 4-PBA, or 4-PBA alone for 24 h, the mRNA levels of the HK-2 cells were determined with a real-time RT-PCR assay as described in Section 2. GAPDH served as a reference gene. The results represent the means ± SD from 3 experiments (a). The protein level was examined with a Western blot analysis after cells were incubated with CML or CML plus anti-RAGE antibody for 24 h. The histogram represents the means ± SD of the densitometric scans for proteins from 3 experiments, normalized by comparison with *β*-actin and expressed as a percentage of the control (b). The nuclear nSREBP-2 protein level was normalized by comparison with histone-H (c). ^*∗*^ *P* < 0.05, compared with the Ctr; ^#^ *P* < 0.05, compared with CML.

We examined the expression of SREBP-2, HMG-CoAR, LDLr, LXR, and ABCA1 to determine the effects of CML on cholesterol metabolism. A dramatic increase in the levels of SREBP-2 mRNA and protein was detected in CML-treated HK-2 cells (Figures 4(a) and 4(b)), and the SREBP-2 nuclear protein was elevated as well (Figure 4(c)). LDLr is the channel used for cholesterol uptake by tubule cells [22], and HMG-CoAR is the key enzyme for cholesterol synthesis [23]. Both the gene expression and protein levels of these two molecules were increased after CML treatment (Figures 4(a) and 4(b)). These results suggest that CML enhanced cholesterol uptake and synthesis through the activation of SREBP-2 in HK-2 cells. Furthermore, the reduced mRNA and protein expression levels of LXR and ABCA1 suggest that CML weakened LXR-ABCA1-mediated cholesterol efflux in the HK-2 cells. However, the anti-RAGE antibody suppressed the CML-mediated dysregulation of SREBP-2, HMG-CoAR, LDLr, LXR, and ABCA1 (Figures 4(a) and 4(b)). These data suggest that blocking the CML-RAGE pathway could reduce cholesterol uptake and synthesis, increase cholesterol efflux, and thus limit lipid accumulation in HK-2 cells.

### 3.4. CML-Induced ER Stress in HK-2 Cells

**Figure 5:**
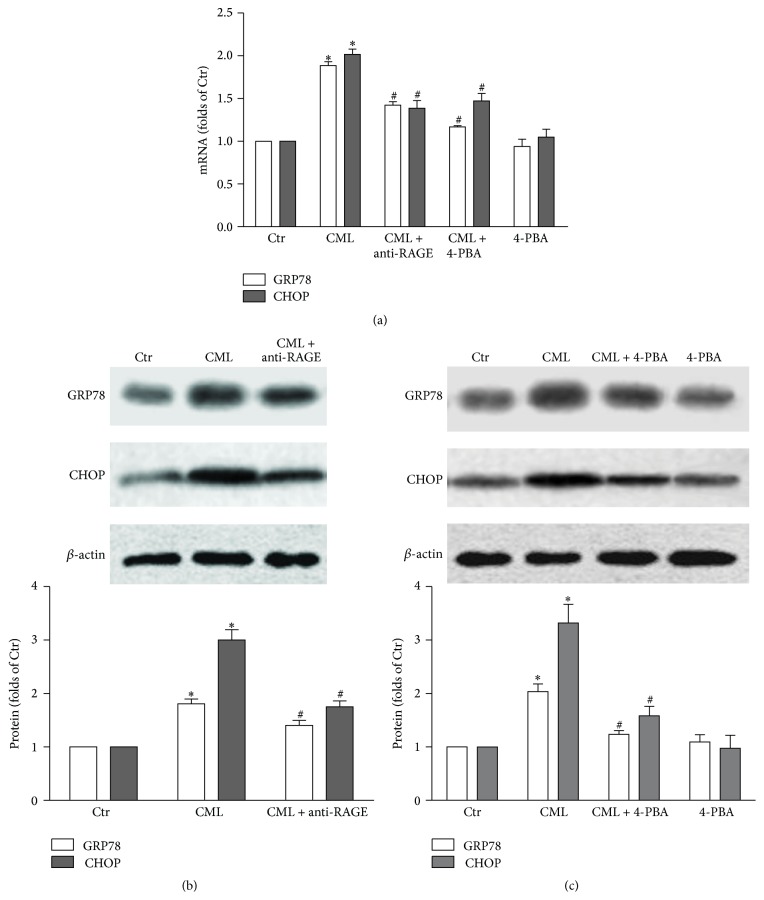
Effects of CML, anti-RAGE antibody, and an ERS inhibitor on mRNA and protein expression levels of GRP78 and CHOP in HK-2 cells. After incubation with CML, CML plus anti-RAGE antibody, CML plus 4-PBA, or 4-PBA alone for 24 h, the mRNA levels of the HK-2 cells were determined with a real-time RT-PCR assay as described in Section 2. GAPDH served as a reference gene. The results represent the means ± SD from 3 experiments (a). The protein level was examined via Western blot analysis after cells were incubated with CML or CML plus anti-RAGE antibody. The histogram represents the means ± SD of the densitometric scans for proteins from 3 experiments, normalized by comparison with *β*-actin and expressed as a percentage of the control (b). The protein level was examined via Western blot analysis after cells were incubated with CML, CML plus 4-PBA, or 4-PBA alone. The histogram represents the means ± SD of the densitometric scans for proteins from 3 experiments, normalized by comparison with *β*-actin and expressed as a percentage of the control (c). ^*∗*^ *P* < 0.05, compared with the Ctr; ^#^ *P* < 0.05, compared with CML.

The GRP-78 is an ER chaperone, the expression of which is increased on exposure to ERS, and CHOP is a transcription factor that is activated during excessive ERS. Our results demonstrated that CML upregulated both the mRNA and protein expression levels of GRP-78 and CHOP, which implies that ERS was triggered by CML in the HK-2 cells. However, both the anti-RAGE antibody and 4-PBA downregulated the expression of GRP-78 and CHOP, suggesting that efficient suppression resulted from either blocking the CML-RAGE pathway or inhibiting ERS (Figures 5(a), 5(b), and 5(c)).

### 3.5. Inhibiting ERS Increases Cell Viability and Reduces Cell Apoptosis

Compared with the HK-2 cells treated with CML alone, the addition of 4-PBA significantly increased cell viability and reduced cell apoptosis in HK-2 cells. However, 4-PBA alone did not affect the cell viability or apoptosis of the HK-2 cells compared with the control (Figures 1 and 2).

### 3.6. Inhibiting ERS Reduces Intracellular Cholesterol Content

4-PBA significantly ameliorated Oil Red O staining and reduced intracellular cholesterol ester in the CML-treated HK-2 cells, suggesting a strong association between ERS and enhanced lipid accumulation in the renal tubules. When cells were treated with 4-PBA alone, there was no obvious change in the intracellular cholesterol content (Figures 3(a) and 3(b)).

### 3.7. Inhibiting ERS Reduces Cholesterol Uptake and Synthesis but Increases Cholesterol Efflux

**Figure 6:**
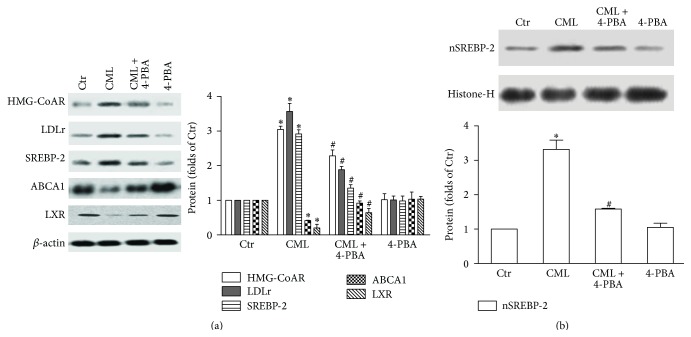
Effects of an ERS inhibitor on the protein expression of HMG-CoAR, LDLr, SREBP-2, ABCA1, and LXR in HK-2 cells. The protein level was examined via Western blot analysis after cells were incubated with CML, CML plus 4-PBA, or 4-PBA alone for 24 h. The histogram represents the means ± SD of the densitometric scans for proteins from 3 experiments, normalized by comparison with *β*-actin and expressed as a percentage of the control (a). Nuclear nSREBP-2 protein level was normalized by comparison with histone-H (b). ^*∗*^ *P* < 0.05, compared with the Ctr; ^#^ *P* < 0.05, compared with CML.

4-PBA significantly reduced both the precursor and mature forms of SREBP-2 and downregulated the CML-induced increase in the mRNA and protein expression levels of HMG-CoAR and LDLr in HK-2 cells. In addition, the mRNA and protein levels of ABCA1 and LXR were elevated by 4-PBA in the CML-treated HK-2 cells. Compared with the control, 4-PBA alone had no effect on the expression of these molecules (Figures 4(a), 6(a), and 6(b)). These results suggest that ERS-related renal tubular foam cell formation is due to increased cholesterol uptake and synthesis and reduced cholesterol efflux.

## 4. Discussion

Initially, renal lipid deposition was found only in acute kidney injury [24, 25], but in recent years an increasing number of studies have demonstrated that there is lipid accumulation in the kidneys of diabetic rodents [26–28]. In 2013, Herman-Edelstein et al. confirmed that lipid deposition also takes place in the kidneys of diabetic patients [29], making fatty kidney research a hot topic in the field. Moreover, exploring the mechanisms underlying lipid accumulation has become a pressing issue. Our previous study showed that there are excessive amounts of lipid droplets in type 2 diabetic rats, especially in the tubules, which is associated with tubule injury [18]. Because the tubules are exposed to large quantities of CML in diabetic patients, we hypothesized that the accumulated CML in diabetic kidneys may be related to tubular foam cell formation.

To gain insight into the mechanisms of renal tubular lipid accumulation, we treated HK-2 cells with CML. Using Oil Red O staining, the present study demonstrates that CML causes lipid accumulation in HK-2 cells, which was confirmed by an intracellular cholesterol ester quantitative assay. Next, we examined the effects of CML on the expression levels of SREBP-2, HMG-CoAR, LDLr, LXR, and ABCA1, and we also used an anti-RAGE antibody to block the CML-RAGE pathway to determine the effects of CML on cellular cholesterol uptake, synthesis, and efflux. All of these data suggested that CML disrupts cholesterol metabolism in HK-2 cells by activating SREBP-2 and LXR, followed by increased HMG-CoAR-mediated cholesterol synthesis, LDLr-mediated cholesterol uptake, and reduced ABCA1-mediated cholesterol efflux, ultimately causing lipid accumulation in the HK-2 cells.

It is well known that the ERS response is an adaptive mechanism by which cells react to perturbations in ER homeostasis through the upregulation of ER-resident chaperons, such as GRP78. When ER function is severely impaired, this organelle emits apoptotic signals, for example, through the activation of CHOP [30]. CHOP represses the normal unfolded protein response and plays an important role in ERS-induced apoptotic cell death. Our results showed that CML increased the expression levels of GRP78 and CHOP, suggesting that CML triggered ER stress in the HK-2 cells, thus resulting in decreased cell viability and increased cell apoptosis.

When we used 4-PBA to inhibit ERS, we found that 4-PBA downregulated the CML-induced upregulation of SREBP-2, nSREBP-2 HMG-CoAR, and LDLr in HK-2 cells, suggesting that inhibiting ERS reduces the abnormal cellular cholesterol uptake and synthesis associated with CML. Furthermore, we also found that 4-PBA upregulated the expression of LXR and ABCA1, improving the CML-impaired cellular cholesterol efflux. All of these resulted in reduced intracellular cholesterol content after 4-PBA treatment in HK-2 cells.

Although the mechanisms underlying the activation of SREBP-2 and LXR by ERS remain unclear, this activation might result from the reduced retention of SREBP-2 in the ER and the enhanced translocation of this protein from the ER to the Golgi [31]. Then, after cleavage in the Golgi, the activated nSREBP-2 is transferred to the nucleus to promote the transcription of HMG-CoAR and LDLr. Some other studies have shown that intracellular lipid accumulation plays an important role in triggering ERS [32, 33], and this accumulation can be alleviated by LXR-induced lipid efflux [34]. However, the mechanism by which ERS affects LXR-related gene transcription is still unclear, and our subsequent work will focus on this issue.

In conclusion, this study demonstrates that CML increases HMG-CoAR-mediated cholesterol synthesis and LDLr-mediated cholesterol uptake and reduces ABCA1-mediated cholesterol efflux though ERS, which ultimately causes lipid accumulation in HK-2 cells. Inhibiting ERS is a potential novel approach to treating the renal tubule lipid accumulation caused by AGEs (CML).

## Figures and Tables

**Table 1:** The primers for real-time RT-PCR.

Gene	Primers
HMG-CoAR	5′-TACCATGTCAGGGGTACGTC-3′ sense
	5′-CAAGCCTAGAGACATAATCATC-3′ antisense

LDLr	5′-CCAAATGATGCCACTTCCC-3′ sense
	5′-ATCCCATCCCAACACACAC-3′ antisense

SREBP-2	5′-CCCTTCAGTGCAACGGTCATTCAC-3′ sense
	5′-TGCCATTGGCCGTTTGTGTC-3′ antisense

ABCA1	5′-CAATCTCACCACTTCGGTCTCCA-3′ sense
	5′-CTCTTCTCATCACTTTCCTCGCC-3′ antisense

LXR	5′-TGAAGAAACTGAAGCGGCAAGA-3′ sense
	5′-CAGAAGCATCACCGTGACTCGA-3′ antisense

GRP78	5′-AAAGCTAAGAAGAAGGAACTGGAAG-3′ sense
	5′-CAACTCATCTTTTTCTGCTGTATCC-3′ antisense

CHOP	5′-CTTGACCCTGCTTCTCTGGCTT-3′ sense
	5′-TTCCGTTTCCTGGTTCTCCCTT-3′ antisense

GAPDH	5′-TGTTGCCATCAACGACCCCTT-3′ sense
	5′-CTCCACGACATACTCAGCA-3′ antisense
